# Inflammatory Biomarkers and ECG Repolarization Metrics: Sudden Death in Heart Failure With Reduced Ejection Fraction

**DOI:** 10.1016/j.jacadv.2026.102922

**Published:** 2026-06-17

**Authors:** Haran Yogasundaram, Wendimagegn Alemayehu, Eric Ly, Christopher R. deFilippi, Christopher M. O’Connor, Tracy Temple, Roopinder K. Sandhu, Adriaan A. Voors, Cynthia M. Westerhout, Paul W. Armstrong

**Affiliations:** a Canadian VIGOUR Centre, University of Alberta, Edmonton, Alberta, Canada; b Inova Heart and Vascular Institute, Falls Church, Virginia, USA; c Department of Cardiology, University Medical Center of Groningen, University of Groningen, Groningen, the Netherlands

**Keywords:** heart failure with reduced ejection fraction, inflammatory biomarkers, sudden cardiac death, VICTORIA trial

## Abstract

**Background:**

Sudden cardiac death (SCD) is a leading cause of mortality in heart failure with reduced ejection fraction (HFrEF). Although prolonged corrected QT (QTc) interval is associated with inflammation and SCD, the association between inflammatory biomarkers, electrocardiogram (ECG) repolarization metrics and SCD remains unclear in HFrEF.

**Objectives:**

The objective of the study was to assess the relationship between ECG repolarization intervals, inflammation, and SCD in HFrEF.

**Methods:**

We evaluated 4,391 patients with HFrEF and recent decompensation in the VICTORIA trial who had core lab-adjudicated ECGs and baseline biomarkers. We assessed whether interleukin-6, growth differentiation factor-15, and high-sensitivity C-reactive protein (hsCRP) were associated with SCD and an association between QTc interval prolongation and SCD. JTc interval was also included and defined as QTc minus the QRS duration. Cox proportional hazards and linear regression models were used.

**Results:**

JTc interval (HR: 0.97 per 10 ms; 95% CI: 0.94-1.00; *P* = 0.043), but not QTc interval, was associated with SCD. All 3 biomarkers were associated with increased SCD risk in univariable analysis, but only hsCRP remained significant in multivariable models (HR: 1.20 per doubling; *P* < 0.001). Growth differentiation factor-15 was independently associated with QTc and JTc intervals. When each inflammatory biomarker was added separately to the covariate-adjusted JTc interval model, hsCRP provided the greatest prognostic value (16% new information; *P* < 0.001).

**Conclusions:**

Inflammatory biomarkers, particularly hsCRP, are independently associated with SCD in patients with HFrEF and provide prognostic value beyond ECG parameters. These findings suggest that inflammation likely contributes to SCD risk through pathways distinct from repolarization prolongation. Biomarkers such as hsCRP may be useful to incorporate into risk stratification.

Heart failure (HF) with reduced ejection fraction (HFrEF) is associated with high cardiac mortality including sudden cardiac death (SCD). Predicting which patients are at a greatest risk of SCD is challenging.^1^ Prolongation of the corrected QT (QTc) interval on electrocardiogram (ECG) is a known predictor of SCD both in the general population and among those with HF.^2^, ^3^, ^4^ Systemic inflammation has also been linked to increased risk of QT prolongation and SCD.^5^^,^^6^ Increasing data from clinical studies suggests that the inflammatory cytokine interleukin (IL)-6 is associated with QT-interval prolongation and arrhythmogenesis.^7^, ^8^, ^9^, ^10^ Growth differentiation factor (GDF)-15, a cytokine secreted by cardiac myocytes in response to inflammation, is also associated with an increased risk of SCD in myocardial infarction and cardiomyopathy.^11^^,^^12^ High-sensitivity C-reactive protein (hsCRP), a nonspecific inflammatory biomarker, has also been associated with prolonged QTc and SCD in patients with coronary artery disease (CAD).^13^ However, the relationship between these inflammatory biomarkers, QTc prolongation, and SCD in the HF population is unclear. The JTc interval, calculated by subtracting the QRS duration from the QTc interval, is more predictive of mortality than QTc and is particularly important in the HF population with a large burden of conduction disease.^14^^,^^15^

The VICTORIA (VerICiguaT Global Study in Subjects With Heart Failure With Reduced Ejection Fraction; NCT02861534) was an international, multicentre study of 5,050 patients with recent worsening, that assessed the efficacy of vericiguat patients with chronic HFrEF and recent HF decompensation.^16^ A subset of this large patient cohort with ECG assessment experienced 824 cardiovascular (CV) deaths during a median of 13.7 months; 26% of these deaths were SCD.^17^ Because these participants had detailed biomarker and core lab-adjudicated ECG data, they offer opportunities for novel insights into the relationship between inflammatory biomarkers, QT intervals, and SCD. We hypothesized that elevated levels of the inflammatory biomarkers IL-6, GDF-15, and hsCRP would be independently associated with the risk of SCD and provide incremental risk stratification of SCD beyond ECG parameters alone.^17^

## Methods

### Study population

VICTORIA was a prospective, double-blind, multinational, randomized-controlled trial of patients with chronic, worsening HF (recent HF hospitalization or use of intravenous diuretics), reduced left ventricular ejection fraction (LVEF) (<45%), and elevated N-terminal pro–B-type natriuretic peptide (NT-proBNP).^16^^,^^18^ The present substudy population was comprised of 4,391 participants with valid baseline QT interval and biomarker data from the original VICTORIA cohort of 5,050 patients. A 30-day screening period was used to ensure clinical stability at the time of baseline assessment.

### Ethics statement

The protocol was approved by participating ethics committees and Institutional Review Boards and patients provided written informed consent. The study complied with the Declaration of Helsinki.

### Baseline data and outcomes

Baseline demographic, historical, and electrolyte data were collected at participating sites. Baseline ECGs were acquired at participating sites and interpreted by independent, blinded reviewers at a centralized core laboratory at the Canadian VIGOUR Centre in Edmonton, Canada, as previously described.^17^ ECG interpretation was performed using guideline recommendations.^19^ QT interval was preferentially measured in lead II, but V_5_ or V_6_ were used if lead II was not suitable. The tangential line method was used where appropriate.^20^ In patients with atrial fibrillation (n = 1,992), as rhythm strips were not routinely collected, an average of 3 QT measurements were made and the preceding R-R intervals were used to estimate rate. The QT interval was corrected for rate using the Bazett and Fridericia formulas.^21^^,^^22^ To adjust for an increase in the QT interval due to conduction disease, the JTc interval was calculated as the difference between the QTc (Bazett) and QRS duration.^14^

Serum samples were collected from participants at baseline and stored at −80 °C before being thawed for analysis. IL-6 and hsCRP (Siemens Diagnostics) were measured at baseline using standard techniques at the University of Maryland (Baltimore, Maryland).^23^ Subsequently, GDF-15 and high-sensitivity cardiac troponin (Cobas e602; Roche Diagnostics) were measured using standard techniques at the Inova Heart and Vascular Institute (Falls Church, Virginia).

The primary clinical outcome evaluated in this substudy was SCD. SCD was defined as occurring unexpectedly and not following an acute myocardial infarction. It included: 1) death witnessed and without new/worsening symptoms; 2) death witnessed within 60 minutes of new or worsening cardiac symptoms; 3) death witnessed and attributed to an identified arrhythmia or unwitnessed but detected on an implantable cardioverter-defibrillator (ICD); 4) death after unsuccessful resuscitation from cardiac arrest; or 5) unwitnessed death in a participant seen alive and clinically stable within 24 hours without evidence to support a non-CV etiology.^16^ All outcomes were centrally adjudicated by a blinded clinical events committee. The median follow-up for mortality was 13.7 months.

### Statistical analyses

Categorical demographic and clinical data and ECG measurements are presented as counts and percentages, whereas biomarker data and continuous ECG measurements are presented as medians (25th-75th percentiles). A series of Cox proportional hazards models was used to examine associations between biomarkers, ECG measurements, and SCD. First, each of the biomarkers and the ECG measurements were modeled individually alongside prespecified covariates. (models 1-6) These covariates may be related to QTc interval or risk of SCD and included the Meta-Analysis Global Group in Chronic Heart Failure (MAGGIC) risk score, NT-proBNP, index clinical event at randomization, ICD usage, and history of CAD. Observations with missing data in covariates were not imputed; thus, participants were excluded from the adjusted analyses if these data were not available. Subgroup analyses of the relationship between QTc or JTc intervals and SCD were conducted according to ICD status at randomization and QRS length (≥120 ms, <120 ms). HRs and 95% CIs for the ECG measurements were adjusted for NT-proBNP, MAGGIC risk score, index event, CAD (and ICD use when examining the QRS length subgroup), and the *P* values for the interactions are reported.

Second, each of the 3 biomarkers was added 1 by 1 to the ECG measurement model and assessed for its marginal added predictive value (models 7-9). Finally, a model consisting of all biomarkers, ECG measurement, and the adjustment covariates was fitted to assess the relative contributions of the biomarkers (model 10). Changes in likelihood ratio chi-square and Harrell C-index were used to evaluate additive predictive value. Modeling assumptions of linearity of the continuous measures was tested using restricted cubic spline and logarithmic transformation or piecewise linear regression where appropriate. Associations with SCD were estimated and reported as adjusted HR and 95% CI.

To examine the relationship between the inflammatory biomarkers and ECG measurements, linear regression was used and included adjustment for the covariates described previously and additionally for calcium, potassium, chloride, and amiodarone (a known QT-prolongation medication). These relationships were further assessed for linearity and were flexibly described using the restricted cubic spline regression.

All analyses were performed at the Canadian VIGOUR Center, University of Alberta (Edmonton, Canada) using SAS (version 9.4; SAS Institute Inc) and R (version 4.3.2; R Foundation for Statistical Computing). A 2-sided test result with *P* < 0.05 was considered statistically significant. As our study was exploratory in nature, correction for multiple testing was not performed.

## Results

### Participant characteristics, and ECG and inflammatory biomarkers

Of the 4,391 patients in this substudy of the VICTORIA trial with valid baseline QT interval and IL-6 data, the median age was 69 years, 24% were females, 29% had ICDs, and 15% were taking amiodarone (Table 1). Relative to survivors, those with SCD had lower ICD use, more frequently had wider QRS intervals, and shorter JTc intervals. The median QTc (Bazett) was similar in patients with and without SCD: 459 ms and 457 ms, respectively (Table 2). Similarly, QTc (Fridericia) was 438 ms and 442 ms. However, baseline IL-6, GDF-15, and hsCRP levels were all significantly elevated in those with SCD as compared to those patients without SCD (Table 1). We also evaluated high-sensitivity cardiac troponin levels (mgs/L), which were similarly elevated in patients with SCD (42 [(26-63]); vs 30 (19-49) overall and 27 (18-43) in patient survivors (Table 1).

**Table 1:** Selected Participant Demographics, Electrolytes, QT-Prolonging Medications, and ECG Measurements at Randomization in the Overall Study Cohort and by Mode of Death

	Overall (N = 4,391)	SCD (n = 198)	No Sudden Cardiac Death				*P* Value^b^
			Total (n = 4,193)	CV Death (n = 559)	Non-CV Death (n = 175)	No Death (n = 3,459)	
Age, y (n = 4,391)	69 (60, 77)	69 (61, 76)	69 (60, 77)	71 (63, 78)	73 (65, 80)	68 (59, 76)	0.9
Female (n = 4,391)	1,061 (24%)	44 (22%)	1,017 (24%)	119 (21%)	44 (25%)	854 (25%)	0.5
Index event (n = 4,391)							0.5
HF hosp. 3-6 mo	792 (18%)	37 (19%)	755 (18%)	89 (16%)	35 (20%)	631 (18%)	
HF hosp. within 3 mo	2,890 (66%)	135 (68%)	2,755 (66%)	392 (70%)	124 (71%)	2,239 (65%)	
IV diuretic for HF within 3 mo	709 (16%)	26 (13%)	683 (16%)	78 (14%)	16 (9.1%)	589 (17%)	
Heart rate, beats/m (n = 4,367)	72 (63, 83)	72 (62, 84)	72 (63, 83)	74 (65, 84)	73 (63, 84)	72 (63, 82)	0.7
History of CAD (n = 4,391)	2,451 (56%)	127 (64%)	2,324 (55%)	364 (65%)	101 (58%)	1,859 (54%)	0.016
eGFR, mL/min/1.73 m^2^ (n = 4,342)	58 (41, 76)	55 (37, 74)	58 (41, 76)	50 (33, 69)	42 (29, 62)	59 (43, 78)	0.2
NT-proBNP, pg/mL (n = 4,216)	2,819 (1,560, 5,333)	4,587 (2,536, 8,843)	2,768 (1,534, 5,209)	5,058 (2,652, 9,679)	5,704 (2,982, 9,962)	2,466 (1,413, 4,462)	<0.001
Potassium, mEq/L (n = 4,311)	4.5 (4.2, 4.8)	4.5 (4.1, 4.8)	4.5 (4.2, 4.8)	4.5 (4.1, 4.8)	4.5 (4.0, 4.9)	4.5 (4.2, 4.8)	0.4
Calcium, mEq/L (n = 4,348)	9.3 (9.0, 9.6)	9.3 (9.0, 9.6)	9.3 (9.0, 9.6)	9.3 (8.9, 9.6)	9.2 (8.8, 9.5)	9.3 (9.0, 9.7)	0.2
Chloride, mEq/L (n = 4,348)	100.0 (97.0, 102.0)	99.0 (95.0, 101.0)	100.0 (97.0, 102.0)	98.0 (95.0, 101.0)	98.0 (95.0, 101.0)	100.0 (97.0, 102.0)	0.006
Amiodarone (n = 4,391)	648 (15%)	35 (18%)	613 (15%)	93 (17%)	36 (21%)	484 (14%)	0.2
ICD (n = 1,265)	1,266 (29%)	41 (21%)	1,225 (29%)	212 (38%)	53 (30%)	960 (28%)	0.010
MAGGIC risk score (n = 4,311)	24 (19, 28)	25 (21, 30)	24 (19, 28)	27 (23, 32)	29 (24, 31)	23 (19, 27)	0.005
ECG measurements							
QRS duration, ms (n = 4,387)	111 (91, 143)	121 (96, 149)	110 (90, 142)	120 (96, 149)	120 (94, 147)	108 (90, 141)	0.004
QRS ≥120 ms (n = 4,387)	1,915 (44%)	104 (53%)	1,811 (43%)	280 (50%)	89 (51%)	1,442 (42%)	0.010
QT interval, ms (n = 4,391)	414 (379, 454)	411 (375, 463)	414 (379, 454)	418 (380, 458)	415 (373, 465)	413 (379, 452)	0.8
Corrected QT, Bazett, ms (n = 4,391)	457 (427, 490)	459 (427, 487)	457 (427, 490)	466 (438, 497)	460 (430, 493)	455 (425, 488)	>0.9
Corrected QT, Fridericia, ms (n = 4,391)	442 (413, 474)	438 (411, 473)	442 (413, 474)	450 (421, 480)	447 (411, 477)	441 (411, 473)	0.8
JTc interval^a^, ms (n = 4,387)	340 (312, 369)	335 (309, 366)	340 (313, 369)	342 (315, 370)	342 (308, 371)	339 (313, 369)	0.081
Biomarkers							
IL-6, pg/mL (n = 4,391)	7 (5, 11)	9 (6, 15)	7 (5, 11)	10 (6, 17)	9 (6, 16)	6 (4, 10)	<0.001
GDF-15, pg/mL (n = 4,160)	3,046 (1,919, 5,155)	3,844 (2,510, 6,300)	3,009 (1,904, 5,097)	5,043 (2,914, 8,338)	5,058 (3,031, 7,915)	2,762 (1,795, 4,494)	<0.001
hsCRP, mg/L (n = 4,282)	4 (1, 9)	7 (3, 16)	4 (1, 9)	7 (2, 15)	6 (2, 15)	3 (1, 8)	<0.001
hsTnT, ng/L (n = 4,363)	30 (19, 49)	42 (26, 63)	29 (19, 48)	45 (27, 72)	44 (27, 77)	27 (18, 43)	<0.001

CAD = coronary artery disease; CV = cardiovascular; ECG = electrocardiogram; eGFR = estimated glomerular filtration rate; GDF = growth differentiation factor; HF = heart failure; hosp = hospitalization; hsCRP = high-sensitivity C-reactive protein; hsTnT = high-sensitivity cardiac troponin; ICD = implantable cardioverter-defibrillator; IL = interleukin; IV = intravenous; MAGGIC = Meta-Analysis Global Group in Chronic Heart Failure; NT-proBNP = N-terminal pro–B-type natriuretic peptide; SCD = sudden cardiac death.

a JTc interval = Bazett corrected QT – QRS duration.

b *P* values are for the comparison of SCD vs no SCD groups.

**Table 2:** Adjusted Associations of ECG Parameters (Including Q-Wave) and Inflammatory Biomarkers at Randomization With Sudden Cardiac Death

	Model	Number of Patients	SCD (n = 198)	No SCD (n = 4,193)	Adjusted HR (95% CI)^a^	*P* Value
ECG parameter						
Corrected QT intervals (per 10 ms)						
Bazett	1	4,154	459 (427, 487)	457 (427, 490)	1.00 (0.97-1.03)	0.84
Fridericia	2	4,154	438 (411, 473)	442 (413, 474)	0.99 (0.96-1.02)	0.67
JTc interval	3	4,150	335 (309, 366)	340 (313, 369)	0.97 (0.94-1.00)	0.043
Biomarkers						
IL-6 (per doubling)	4	4,154	9 (6, 15)	7 (5, 11)	1.23 (1.08-1.39)	0.0013
GDF-15 (per doubling)	5	3,952	3,844 (2,510, 6,300)	3,009 (1,904, 5,097)	1.21 (1.03-1.42)	0.019
hsCRP (per doubling)	6	4,061	7 (3, 16)	4 (1, 9)	1.21 (1.12-1.13)	<0.001

Abbreviations as in Table 1.

a Models for each ECG measurement or inflammatory biomarker were separately adjusted for NT-proBNP, MAGGIC score, index event, ICD use, history of CAD.

### Relative associations with SCD

After adjustment for relevant participant characteristics, QTc interval measurements were not associated with SCD (Table 2) (models 1-2). A longer JTc interval (per 10 ms) was associated with a declining hazard of SCD (model 3: adjusted HR, 0.97 per 10 ms; 95% CI, 0.94-1.00; *P* = 0.043). When the relationships of the ECG measurements with SCD were examined for heterogeneity according ICD use at randomization and QRS duration, no significant interactions with QTc or JTc intervals were observed (Supplemental Table 1). Higher values (per doubling) of all inflammatory biomarkers were associated with excess SCD (Central Illustration and Table 2: models 4-6).

**Central Illustration:**
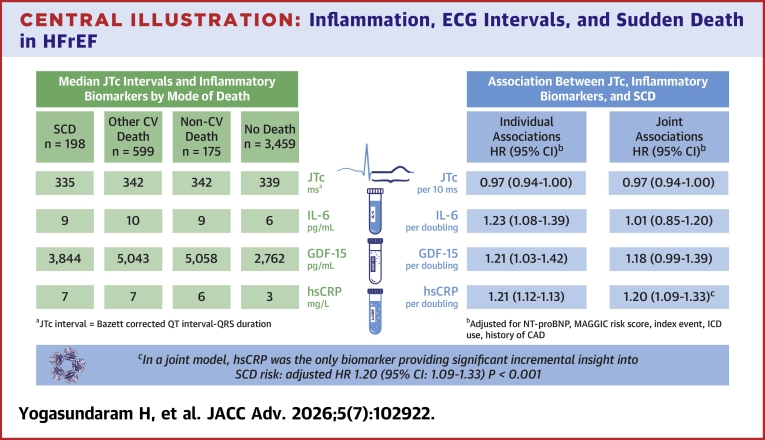
**Inflammation, ECG Intervals, and Sudden Death in HFrEF** CAD = coronary artery disease; CV = cardiovascular; ECG = electrocardiogram; GDF = growth differentiation factor; HFrEF = heart failure with reduced ejection fraction; hsCRP = high-sensitivity C-reactive protein; ICD = implantable cardioverter-defibrillator; IL = interleukin; MAGGIC = Meta-Analysis Global Group in Chronic Heart Failure; NT-proBNP = N-terminal pro–B-type natriuretic peptide; SCD = sudden cardiac death.

Given the association between JTc interval and SCD, it was jointly modeled with each inflammatory biomarker and the other covariates to evaluate the added predictive value of the individual biomarkers (Central Illustration and Table 3: models 7-10). JTc interval continued to be associated with declining hazard of SCD and, individually, each biomarker retained its positive association with SCD (models 7-9). However, when all biomarkers were included in the model (model 10), hsCRP was the only inflammatory biomarker to retain a significant association with SCD (adjusted HR: 1.20 per doubling; 95% CI 1.09-1.33; *P* < 0.001). HsCRP also contributed the highest fraction of new information when added to both the individual (model 9: 26.0%) and overall models (model 10: 16.0%).

**Table 3:** Adjusted Associations of JTc Interval and the Inflammatory Biomarkers With Sudden Cardiac Death

	Adjusted^a^ HR (95% CI)	*P* Value	bLR Chi-Square	C-Statistic	Change in LR Chi-Square	Fraction of New Information From Biomarker
Model 7: JT interval + IL-6			86.54	0.69	9.70 (*P* = 0.0012)	11.2%
JTc interval (per 10 ms)	0.97 (0.94-1.00)	0.038				
IL-6 (per doubling)	1.23 (1.09-1.39)	0.0012				
Model 8: JT interval + GDF-15			82.23	0.70	5.40 (*P* = 0.010)	6.6%
JTc interval (per 10 ms)	0.97 (0.94-1.00)	0.036				
GDF-15 (per doubling)	1.24 (1.05-1.45)	0.010				
Model 9: JT interval + hsCRP			103.85	0.71	27.01 (*P* < 0.001)	26.0%
JTc interval (per 10 ms)	0.97 (0.94-1.00)	0.031				
hsCRP (per doubling)	1.21 (1.12-1.32)	<0.001				
Model 10: JT interval + all biomarkers^c^			105.97	0.72		
JTc interval (per 10 ms)	0.97 (0.94-1.00)	0.034				
IL-6 (per doubling)	1.01 (0.85-1.20)	0.92	105.96^d^		0.009 (*P* = 0.92)	0.000%
GDF-15 (per doubling)	1.18 (0.99-1.39)	0.058	104.06^d^		1.92 (*P* = 0.058)	1.8%
hsCRP (per doubling)	1.20 (1.09-1.33)	<0.001	89.06^d^		16.9 (*P* < 0.001)	16.0%

Abbreviations as in Table 1.

a Models for each ECG measurement or inflammatory biomarker were separately adjusted for NT-proBNP, MAGGIC score, index event, ICD use, history of CAD.

b LR chi-square for the model of JTc interval + covariates (a) was 76.84.

c Effective sample size in the parsimonious model was N = 3,867.

d LR chi-square for the model of JTc interval + covariates + biomarkers simultaneously except the corresponding biomarker.

### ECG and inflammatory biomarkers

**Figure 1:**
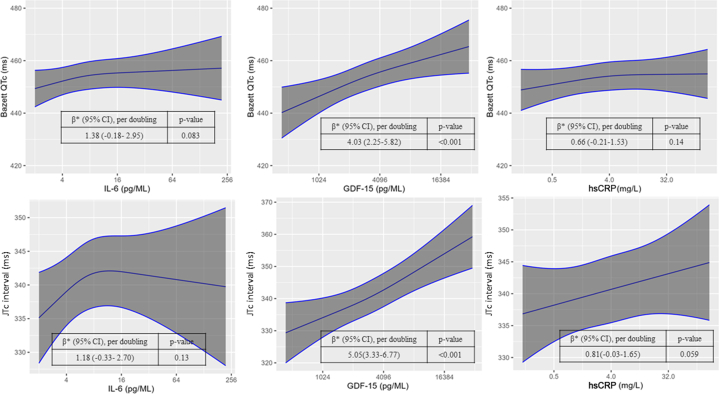
**Continuous Relationship of QTc and JT Interval With Inflammatory Biomarkers at Randomization** **∗**β linear coefficients are adjusted for NT-proBNP, MAGGIC risk score, index event, ICD use, CAD, calcium, potassium, chloride, and amiodarone. GDF = growth differentiation factor; hsCRP = high-sensitivity C-reactive protein; IL = interleukin; QTc = corrected QT.

Figure 1 illustrates the relationship between the ECG measurements and each of the inflammatory biomarkers. After multivariable adjustment, only GDF-15 was significantly associated with QTc (Bazett; *P* < 0.001) and JTc (*P* < 0.001) intervals such that with higher GDF-15, both QTc and JT intervals were also longer.

## Discussion

In this large cohort of patients with worsening HF on guideline-directed medical therapy from the VICTORIA trial, we found that the inflammatory biomarkers IL-6, GDF-15, and hsCRP were independently associated with SCD after adjustment for NT-proBNP, MAGGIC risk score, index event, ICD use, CAD, electrolytes, and amiodarone use. In a multivariable analysis with all biomarkers and JTc, hsCRP was an independent predictor of SCD risk (Central Illustration). Although GDF-15 was independently associated with QTc and JTc prolongation, the role of IL-6, GDF-15, and hsCRP in the prediction of SCD appears to be independent of QTc interval prolongation.

In both the general population and patients with torsade-de-pointes, there are data to support the association between the inflammatory cytokine IL-6 and QT-interval prolongation.^7^^,^^9^ In the present study, we found that the association between IL-6 and both QTc and JTc did not persist after multivariable adjustment. The difference in our findings may be due to the nature of our HF population or more comprehensive adjustment of covariates. HsCRP in non-HF populations is associated with prolonged QTc and SCD.^10^^,^^13^ We also noted this association in our study which persisted despite multivariable adjustment. HsCRP was also noted to provide the greatest additional prognostic information beyond JTc interval for the prediction of SCD.

The cytokine GDF-15 is associated with an increased risk of SCD in myocardial infarction and cardiomyopathy, but, to our knowledge, its association with QTc has not been previously evaluated.^11^^,^^12^ We found that GDF-15 was associated with both QTc and JTc intervals and that this relationship persisted even after multivariable adjustment. Animal data suggest that GDF-15 has a protective, antihypertrophic, and antiapoptotic role in the heart.^24^ Patients with GDF-15 knockout variants have prolonged QTc intervals, suggesting that the loss of the anti-inflammatory effects of GDF-15 may contribute to prolonged QTc.^25^ In patients with HF, elevated GDF-15 levels in response to cardiac stress may be a compensatory response to an underlying proinflammatory state associated with prolonged QTc.

SCD remains a significant issue in HF patients treated with contemporary medical therapy and was the cause of death in 27% of a pooled data set of 11,007 HF patients (any LVEF) from the Dapagliflozin and Prevention of Adverse Outcomes in Heart Failure and Dapagliflozin Evaluation to Improve the Lives of Patients With Preserved Ejection Fraction Heart Failure trials.^26^ In a study of 398 patients with HFrEF, QTc was independently associated with increased all-cause death, cardiac death, and SCD in a multivariable analysis that included B-type natriuretic peptide and LVEF.^4^ Although the association between IL-6 and hsCRP, with all cause and CV death, the proportion of SCD risk that is mediated via the prolongation of QTc and JTc intervals is unclear.^27^

In our study, we noted that an increase in JTc was paradoxically associated with a decreased risk of SCD (HR, 0.97 per 10 ms increase; 95% CI: 0.94-1.00; *P* = 0.043). As JTc is calculated by subtracting the QRS duration from the QTc interval, we postulate that this finding may be attributable to an overcorrection for prolonged QRS duration. Hence, for a given QTc interval, a shorter JTc interval would be observed in patients with a longer QRS duration due to bundle branch block or intraventricular conduction delay. The development of QRS prolongation in these patients likely portends risk that exceeds the increased risk attributable to the increase in QT interval. Indeed, in other studies of HF, increasing QRS duration is associated with increased mortality.^28^, ^29^, ^30^, ^31^ This novel finding suggests that conventional JT correction in the setting of a prolonged QRS may overcorrect for SCD risk.

### Study Limitations

There are some limitations and strengths that deserve consideration. First, although adjustments were made including baseline HF risk, amiodarone, and electrolytes, other potential confounders could remain including information on shocks received by patients with ICD therapy. Second, a single ECG was used for measurement of QT interval, and may have overlooked dynamic changes. However, this measurement of QT interval is more likely to represent measures acquired in clinical practice when ECGs are obtained intermittently.

Given the absence of routine rhythm recordings we cannot be certain of the accuracy of our measurements in atrial fibrillation. Our findings acquired from a clinical trial should be considered hypothesis generating, and their generalizability requires confirmation in other HF cohorts. Strengths of our study include the large, event rich population and the selection of independently adjudicated and unbiased measures.

## Conclusions

Inflammatory biomarkers, particularly hsCRP, provide significant prognostic information for predicting the risk of SCD in high-risk patients with worsening HF. Although GDF-15 was independently associated with QTc and JTc prolongation, the predictive role of inflammatory biomarkers appears to be independent of QT interval prolongation. Future research should explore whether targeted anti-inflammatory interventions can reduce SCD risk in this high-risk population.

Perspectives **COMPETENCY IN MEDICAL KNOWLEDGE:** This study identifies systemic inflammatory biomarkers as predictors of SCD in HFrEF. Clinicians should note that hsCRP in particular provides independent prognostic value beyond traditional ECG parameters. Integrating hsCRP into clinical assessments allows for improved risk stratification, helping caregivers identify high-risk patients who may require intensified monitoring or more aggressive primary prevention strategies.

**TRANSLATIONAL OUTLOOK:** This research suggests that inflammatory biomarkers markers are relevant mediators of SCD. Although GDF-15 correlates with QT interval prolongation, the SCD risk conferred by inflammation is largely independent of the corrected QT interval, suggesting a distinct pathophysiological pathway. Future translational efforts should investigate whether these biomarkers can further enhance traditional risk stratification, such as assisting in the evaluation of which patients would benefit from ICD therapy, or potentially as therapeutic targets using specific anti-inflammatory medications.

## Funding support and author disclosures

Funding for the VICTORIA trial was provided by 10.13039/100009947Merck Sharp & Dohme LLC, a subsidiary of 10.13039/100004334Merck & Co., Inc., Rahway, NJ, USA and Bayer AG, Wuppertal, Germany. Dr deFilippi reports research funding to Inova Heart and Vascular Institute from 10.13039/100014386Abbott Diagnostics, 10.13039/100016545Roche Diagnostics, 10.13039/501100011699Siemens Healthineers, and Ortho Diagnostics and consults for FujiRebio, Roche Diagnostics, Siemens Healthineers, and Ortho Diagnostics. Dr O’Connor has received research funding from Merck and consulting fees from Bayer, Dey LP, and Bristol-Myers Squibb Foundation. Dr Sandhu has been on speakers’ bureau for/received honoraria from Servier, Bristol-Myers Squibb, and Pfizer. Dr Voors reports research grants from 10.13039/100001003Boehringer Ingelheim and Roche Diagnostics; consulting fees from Merck, Bayer, Anacardio, AstraZeneca, Boehringer Ingelheim, BMS, Corteria, Cytokinetics, Eli-Lilly, Moderna, Novartis, NovoNordisk, Roche Diagnostics, and Tectonic. Dr Westerhout has served as a consultant for Bayer Canada and Boehringer Ingelheim. Dr Armstrong has received grants and personal fees from Merck and Bayer; grants from Sanofi-Aventis Recherche & Development, Boehringer Ingelheim, and CSL Limited; and personal fees from AstraZeneca and Novartis. All other authors have reported that they have no relationships relevant to the contents of this paper to disclose.
