# Supplementary material for: Inflammatory Biomarkers and ECG Repolarization Metrics: Sudden Death in Heart Failure With Reduced Ejection Fraction
Source: JACC Adv. 2026 Jun 17;5(7):102922. doi: 10.1016/j.jacadv.2026.102922 (PMC13292687; doi:10.1016/j.jacadv.2026.102922)
Supplement: Supplemental Table 1 [file mmc1.docx]

**Inflammatory Biomarkers and ECG Repolarization Metrics: Sudden Death Heart Failure with Reduced Ejection Fraction**

**SUPPLEMENTAL APPENDIX**

**Table of Contents**

[**Supplemental Table 1.** Subgroup Analyses by ICD and QRS Duration for the Association between Repolarization Measurement and Sudden Cardiac Death 2](#_Toc218785847)

# **Supplemental Table 1.** Subgroup Analyses by ICD and QRS Duration for the Association between Repolarization Measurement and Sudden Cardiac Death

| **Measurement** | **Overall** | | **ICD Subgroups** | | | **QRS Duration** | | |
| --- | --- | --- | --- | --- | --- | --- | --- | --- |
|  | ^a^HR (95% CI) | *P*-value | ICD  (N=1,266) | No ICD  (N=3,124) |  | ≥120 ms  (N=1,915) | <120 ms  (N=2,472) |  |
|  |  |  | ^a^HR (95% CI) | ^a^HR (95% CI) | *P_interaction_* | ^a^HR (95% CI) | ^a^HR (95% CI) | *P_interaction_* |
| **Repolarization Measurement** |  |  |  |  |  |  |  |  |
| Corrected QT intervals  (per 10 ms) |  |  |  |  |  |  |  |  |
| Bazett | 1.00 (0.97-1.03) | 0.84 | 1.02 (0.96-1.08) | 0.99 (0.96-1.02) | 0.39 | 0.98 (0.94-1.02) | 0.99 (0.95-1.03) | 0.86 |
| Fridericia | 0.99 (0.96-1.02) | 0.67 | 1.01 (0.95-1.07) | 0.99 (0.96-1.02) | 0.53 | 0.98 (0.94-1.02) | 0.98 (0.94-1.02) | 0.99 |
| JTc interval | 0.97 (0.94-1.00) | 0.043 | 0.96 (0.90-1.02) | 0.97 (0.94-1.01) | 0.74 | 0.98 (0.94-1.02) | 0.98 (0.94-1.02) | 0.96 |

^a^ In each ECG measurement, the HR were adjusted for NT-proBNP, MAGGIC score, index event, ICD use (for QRS length subgroup analysis), and CAD.

ICD, implantable cardioverter-defibrillator; HR, hazard ratios; CI, confidence intervals; N-terminal pro–B-type natriuretic peptide; MAGGIC, Meta‐Analysis Global Group in Chronic Heart Failure; ICD, implantable cardioverter-defibrillator; CAD, coronary artery disease
